# Scaling out a palliative compassionate community innovation: Nav-CARE

**DOI:** 10.1177/26323524221095102

**Published:** 2022-05-13

**Authors:** Barbara Pesut, Wendy Duggleby, Grace Warner, Sunita Ghosh, Paxton Bruce, Rowena Dunlop, Gloria Puurveen

**Affiliations:** Principal Research Chair, Palliative and End of Life Care, The University of British Columbia, Okanagan Campus, 1147 Research Road, Kelowna, BC V1V 1V7, Canada; Faculty of Nursing, University of Alberta, Edmonton, AB, Canada; School of Occupational Therapy, Dalhousie University, Halifax, NS, Canada; University of Alberta/Alberta Health Services, Edmonton, AB, Canada; The University of British Columbia, Okanagan Campus, Kelowna, BC, Canada; The University of British Columbia, Okanagan Campus, Kelowna, BC, Canada; The University of British Columbia, Okanagan Campus, Kelowna, BC, Canada

**Keywords:** community, hospice, navigation, palliative, volunteer

## Abstract

**Background:**

There is an urgent need for community-based interventions that can be scaled up to meet the growing demand for palliative care. The purpose of this study was to scale out a volunteer navigation intervention called Nav-CARE by replicating the program in multiple contexts and evaluating feasibility, acceptability, sustainability, and impact.

**Methods:**

This was a scale-out implementation and mixed-method evaluation study. Nav-CARE was implemented in 12 hospice and 3 nonhospice community-based organizations spanning five provinces in Canada. Volunteers visited clients in the home approximately every 2 weeks for 1 year with some modifications required by the COVID-19 public health restrictions. Qualitative evaluation data were collected from key informants (*n* = 26), clients/family caregivers (*n* = 57), and volunteers (*n* = 86) using semistructured interviews. Quantitative evaluation data included volunteer self-efficacy, satisfaction, and quality of life, and client engagement and quality of life.

**Findings:**

Successful implementation was influenced by organizational capacity, stable and engaged leadership, a targeted client population, and skillful messaging. Recruitment of clients was the most significant barrier to implementation. Clients reported statistically significant improvements in feeling they had someone to turn to, knowing the services available to help them in their community, being involved in things that were important to them, and having confidence in taking care of their illness. Improvements in clients’ quality of life were reported in the qualitative data, although no statistically significant gains were reported on the quality of life measure. Volunteers reported good self-efficacy and satisfaction in their role.

**Conclusion:**

The feasibility, acceptability, and sustainability of the program were largely dependent on strong intraorganizational leadership. Volunteers reported that their involvement in Nav-CARE enabled them to engage in ongoing learning and have a meaningful and relational role with clients. Clients and families described the positive impact of a volunteer on their engagement and quality of life.

## Background

The compassionate community approach recognizes that it is everyone’s responsibility to care for each other^1^ (p. 7).

Developing and scaling compassionate community interventions are an important strategy for improving the quality of palliative care. However, many innovations do not get beyond the pilot phase; Canada was once referred to as a land of perpetual pilot projects.^2^ Although pilot studies are a necessary first step for any innovation, the more challenging task is moving pilot studies to scale. This process is more complex than simply repeating the pilot in new contexts. Rather, it requires changes in ‘rules, resource flows, cultural beliefs and relationships in a social system at multiple spatial or institutional scales’^3^ (p. 2). Taking pilot studies to scale requires three types of projects: scale-out projects in which the goal is to replicate and disseminate a program; scale-up projects in which the goal is to influence policy and law to better support the program; and scale-deep projects in which the goal is to impact cultural roots through influencing relationships, cultural values, and individual beliefs and values.^3^ The study reported here is a scale-out study of one compassionate community intervention.

Nav-CARE (Navigation: Connecting, Advocating, Resourcing, Engaging) is a social innovation in which experienced, trained, and mentored volunteers provide quality of life (QOL) navigation in the home for adults experiencing declining health. Volunteers are trained to identify the day-to-day challenges persons are experiencing as a result of declining health and to assist with connecting them to persons and community-based resources that can help. Volunteers develop long-term relationships with clients by visiting them regularly to facilitate connection and support. Clients who seek Nav-CARE volunteer services are often those who are at risk for social isolation or who have limited capacity to solve their day-to-day challenges. To date, Nav-CARE has been implemented primarily through community-based hospice palliative care societies. These experienced volunteers are knowledgeable about the losses and realities of living with declining health.

Nav-CARE was designed to meet four emerging directions for palliative care in Canada. First, it seeks to facilitate a palliative approach to care in which persons are identified early in the palliative trajectory so that proactive support can be provided.^4^ Second, it seeks to contribute to the development of volunteer capacity in Canada. Volunteers have a vital role in palliative care, and yet, often their contributions are not maximized.^5^ Third, it seeks to optimize the services and resources that are available to clients in the community. Our research in rural palliative care revealed how difficult it can be for persons living with palliative needs to identify and access resources.^6,7^ Finally, it seeks to provide a practical program to support a public health/compassionate community (PHCCA) approach to care,^8–10^ an approach in which persons are supported in the social aspects of care within their communities.^11^ There has been increasing emphasis on the vital role that communities play in determining the quality of end-of-life care, but there is a need for evidence-based programs that communities can use to realize this ideal. Several innovative programs that use volunteers to support home-based care have been described in the international literature^12–15^; however, to our knowledge, there are no other programs that prepare and use specially trained volunteers to engage in relationally based, QOL navigation.

The evidence base for Nav-CARE was developed over a decade of research which began with ethnographic work in rural palliative care.^6,16^ Community advisory members who were partners in this ethnographic work indicated the need for a service in which persons living with a palliative diagnosis would have a knowledgeable and compassionate individual to accompany them and help them know what was available in the community. The initial step was to develop a set of competencies for navigation in rural palliative care^17^ and a curriculum for navigation education.^18^ Next, pilot studies were conducted in Alberta and British Columbia in which we used a community-based nurse navigator^19^ and then a nurse navigator in partnership with volunteer navigators to provide services to older persons living at home with serious illness.^20–22^ Based on these pilot studies, we further refined the competencies and designed a model in which volunteers, supported by an established organization and knowledgeable volunteer coordinator (VC), formed the backbone of the intervention.

The decision to use volunteers instead of healthcare professionals was based on the following reasons: the healthcare system in Canada already had persons serving in navigation-type roles (e.g. case managers) although their caseloads were typically high (e.g. 100:1); health regions were reluctant to fund additional positions; and many of the tasks that the volunteers performed in our early studies were not within the scope of health or social care providers. These practical tasks were ones that, when left undone, would critically impact client health and healthcare-seeking. We then conducted knowledge translation studies to better understand how the intervention might need to be adapted in diverse Canadian contexts.^23,24^

The scale-out study reported here sought to replicate the program in multiple contexts to develop a more robust evidence-base for the intervention. The objective of this study was to evaluate the feasibility, acceptability, sustainability, and impact of Nav-CARE in 12 hospice palliative care organizations and 3 community-based organizations serving older persons across urban and rural contexts. The evaluative questions were as follows: What factors influenced Nav-CARE feasibility, acceptability, and sustainability across contexts? How effective was the Nav-CARE education and subsequent mentoring in preparing volunteers to be volunteer navigators? What was the impact of the Nav-CARE program on clients and family? What was the impact of being a Nav-CARE volunteer?

## Methods

### Design/settings/definitions

This was a scale-out implementation and mixed-method evaluation study.^25^ Nav-CARE was implemented in 12 hospice palliative care organizations between May 2018 and March 2021: three urban (>100,000 population), eight small urban (10,000–99,000 population), and one rural (<10,000 population). Four of these organizations had residential hospice beds, the remainder were hospice societies whose focus was delivering services within the community. In addition, Nav-CARE was further adapted and implemented in three community-based organizations serving older persons during the same period: a family and community service organization in a small rural location and two urban societies serving seniors. Sites were located in five Canadian provinces. Organizations were recruited through conference presentations, media stories, and word of mouth.

Feasibility was defined by whether the organization could effectively implement the program which included identifying volunteers and providing organization oversight; acceptability was defined by whether the organization could effectively recruit clients to the program and client perceptions of the program.^26^ Sustainability was defined as ‘the ability to maintain programming and its benefits over time’^27^ (p. 4), in this case past the 1-year implementation period.

### Study sample

The study sample consisted of key organizational informants, Nav-CARE volunteers, and Nav-CARE clients and family caregivers. Key informants included organizational leaders [e.g. executive directors (EDs), board members, and VCs] and healthcare partners who worked closely with the organization. Nav-CARE volunteers were experienced hospice volunteers (or equivalent experience) who underwent Nav-CARE training and subsequently provided services to clients. Clients were older persons living with advanced chronic illness in the home who felt they could benefit from the services of a volunteer navigator. Family caregivers were those individuals who primarily took on the responsibility of assisting clients with their needs.

### The Nav-CARE intervention

Nav-CARE was implemented in three steps. First, organizations used the Nav-CARE Implementation Manual to decide whether Nav-CARE was a good fit for their organization and to prepare for implementation. This implementation manual provides six questions for organizations to consider before implementing Nav-CARE and provides step-by-step implementation instructions. Study sites were provided with a stipend of $2500 from the research grant to assist with start-up costs. Second, the VC situated within each organization recruited three to four volunteers who were then provided with 2-day Nav-CARE in-person training led by an experienced nurse navigator. This training covered the following topics: understanding the volunteer navigator role; assessing client and family QOL; advocating for clients and family; facilitating community connections; supporting access to services and resources; and promoting active engagement. Third, clients were recruited and screened by the VC and matched with volunteers. Volunteers visited clients in the home approximately every 2 weeks for 1 year. The research team provided 1-h monthly virtual mentorship sessions for volunteers that included a combination of group discussion and structured education. Group discussion focused on sharing learnings and challenges in this new role; structured education included specialized topics such as conducting life reviews, finding community resources, understanding spirituality, and volunteering during a pandemic.

### Data collection

Data were collected using both questionnaires and semistructured interviews (Table 1). Interviews were audio-recorded, transcribed, and entered into NVivo^QSR^ for analysis. Interview questions were developed using the five Consolidated Framework for Implementation Research (CFIR) domains which were defined in relation to the Nav-CARE intervention in previous work: Nav-CARE intervention characteristics, the outer setting in which Nav-CARE was implemented (e.g. health and community systems), the inner organizational setting where Nav-CARE was implemented, the characteristics of the individuals involved in implementing Nav-CARE, and the process of delivering Nav-CARE.^28^

**Table 1.** Data collection overview.

Baseline	6 months	12 months
Key informants: Interviews of feasibility, acceptability, and sustainability (*n* = 26)		Key informants: Postimplementation interviews of feasibility, acceptability, and sustainability (*n* = 16)
Nav-CARE volunteers: Self-perceived efficacy in navigation questionnaire (*n* = 86/87) Volunteer QOL using SF12v2 (*n* = 86/87)	Nav-CARE volunteers: Self-perceived efficacy in navigation questionnaire (*n* = 50/38/70) Volunteer QOL using SF12v2 (*n* = 49/38/70) Volunteer satisfaction questionnaire. (*n* = 55/38/70) Semi-structured interview (*n* = 58/38/70)	Nav-CARE volunteers: Self-perceived efficacy in navigation questionnaire (*n* = 33/27/62) Volunteer QOL using SF12v2 (*n* = 33/37/62) Volunteer satisfaction questionnaire. (*n* = 32/37/62) Semi-structured interview (*n* = 30/37/62)
Nav-CARE clients: QOL using SF12v2 (*n* = 50/50) Engagement questionnaire (*n* = 50/50)	Nav-CARE client: QOL using SF12v2 (*n* = 28/36) Engagement questionnaire (*n* = 29/36) Semistructured interview (*n* = 32/36)	Nav-CARE client: QOL using SF12v2 (*n* = 27/29) Engagement questionnaire (*n* = 27/29)
Nav-CARE family caregivers: QOL using SF12v2 (*n* = 7/7)	Nav-CARE family caregivers: QOL using SF12v2 (*n* = 3/6) Semistructured interview (*n* = 3/6)	Nav-CARE family caregivers: QOL using SF12v2 (*n* = 3/6)

Volunteer *n* = number of responses/number of volunteers with a client/ all active volunteers at a time point.

The mixed-method data collection strategy was guided by the four research questions:

1. What factors influenced Nav-CARE feasibility, acceptability, and sustainability? Interviews were conducted with key informants from each organization preimplementation and postimplementation (12 months). A semistructured interview guide explored the reasons for developing a Nav-CARE service, the benefits and challenges of implementing, the quality of the implementation tools, the perceived benefits to the society and broader community, and the sustainability of Nav-CARE postresearch. Field notes were written from reports and informal telephone conversations with key informants throughout the duration of the project.
2. How effective is the Nav-CARE education and subsequent mentoring in preparing volunteers to be volunteer navigators? The Nav-CARE training was evaluated through volunteer self-efficacy questionnaires administered postworkshop and 6 and 12 months posttraining. The volunteer self-efficacy in navigation questionnaire contained 32 competency items (a = 0.98) that reflected competencies from the navigation-based volunteer training.^18^ Respondents were asked to report their self-perceived competence on each item using a 6-point Likert-type scale from *not at all confident* (0) to *highly confident* (5). Volunteers also participated in semistructured interviews in which they were asked to describe specific examples of using the competencies.
3. What is the impact of the Nav-CARE program on clients and families? Impact on clients and family was measured through client and family QOL, and client engagement. QOL data were collected from clients and family at baseline and 6 and 12 months using the SF12v2 health survey. This is a widely used and well-validated QOL tool.^29,30^ Client engagement was measured at 6 and 12 months using an engagement questionnaire designed specifically for the Nav-CARE program. The 12-item engagement questionnaire includes items on social support, community connectedness, information about needed resources, and confidence in decision-making. Participants responded to items such as ‘I feel I know the services available in my community to help me’ using a 5-point Likert-type scale from 1 (*none of the time*) to 5 (*all of the time*). Satisfaction with the Nav-CARE intervention was evaluated through semistructured interviews conducted with clients and families at 6 months into the intervention.
4. What is the impact of being a Nav-CARE volunteer? Volunteer impact was measured using QOL and satisfaction. QOL was measured at baseline and at 6 and 12 months using the SF12v2 health survey. Satisfaction was measured at 6 and 12 months using a 43-item Satisfaction Questionnaire (*a* = 0.917) adapted with permission for the Nav-CARE program.^31^ The satisfaction questionnaire asked respondents to indicate their agreement, using a 5-point Likert-type scale (0 = *strongly disagree* to 5 = *strongly agree*), about their satisfaction with orientation (four items), training (eight items), feedback on performance (nine items), communication (seven items), social contact (four items), and value/respect (11 items). Volunteers also participated in a semistructured interview regarding their opinions of the Nav-CARE intervention at 6 and 12 months.

### Data analysis

Data were analyzed using a combination of deductive and inductive methods. The volunteer and client data were coded using the four functions of a Nav-CARE volunteer: connecting, advocating, resourcing, and engaging. Data within those open codes were then coded using an inductive method. The coding steps followed the procedure outlined by Braun and Clarke.^32^ Interviews were transcribed verbatim; investigators familiarized themselves with the data; initial codes were developed and negotiated by two investigators; and then themes were generated, refined, and defined using a reflexive approach. Trustworthiness of data was maintained by transcribing interviews verbatim by a transcriptionist, maintaining an audit trail of analysis decisions, and using participants' words as much as possible. Quantitative data were entered into SPSS, cleaned, and analyzed using descriptive statistics. Mean and standard deviations were reported for the QOL and engagement data for each time point separately. Generalized estimating equation (GEE) method was used to compare the change over time for the engagement data from clients and the QOL components for clients and volunteers. The outcome of interest was continuous; hence, parameter estimates and the corresponding 95% confidence intervals were reported. The GEE method provides robust parameter estimates and standard errors for repeated measures data.

## Findings

Eighty-seven volunteers were trained across the 15 implementation sites. Fifty clients and seven family caregivers received volunteer services and participated in the research (Table 2). Some clients who received services chose not to participate in the research; their numbers were not made available to the research team. Two sites were fully recruited (i.e. each volunteer had at least one client), four sites were unable to recruit clients (two of which were nonhospice sites), and the remaining sites were able to recruit clients for some of their volunteers. Only findings from those volunteers who received clients during the intervention period are reported here. Family caregiver data are not reported because of the small sample size.

**Table 2.** Demographic information of participants.

Participant	Variable	Results
Clients (*n* = 50)	Age	Mean: 71.78 (SD: 12.43) Range: 38–94
	Sex	Female: *n* = 35 (70%) Male: *n* = 13 (26%) Missing: *n* = 2 (4%)
	Number of chronic health conditions (self-identified)	1: *n* = 14 (28%) 2: *n* = 15 (30%) 3: Or greater: *n* = 20 (40%) Missing: *n* = 1 (2%)
	Living arrangements	Home alone: *n* = 29 (58%) Home with family: *n* = 15 (30%) Other (e.g. assisted living): *n* = 5 (10%) Missing: *n* = 1 (2%)
Volunteers (*N* = 87)	Age	Mean: 62.89 Range: 24–82
	Sex	Female: *n* = 76 (87.4%) Male: *n* = 11 (12.6%)
	Years of volunteer experience	0–5 years: *n* = 25(28.7%) 6–10 years: *n* = 18 (20.7%) >10 years: *n* = 43 (49.4%) Missing: *n* = 1 (1%)

Demographic data were not collected from key informants.

Clients who participated in the research identified a number of chronic health conditions that they lived with. These clients were not high users of healthcare services: 60.8% of clients had seen their family physician at least once in the previous month (range: 1–8 times) and 84% had not spent any time in hospital in the last month. Furthermore, although the intervention was targeted toward older persons, organizations felt it was important to extend Nav-CARE to any adult who might require services and so the age range of clients was 38–94. For the seven family caregivers who chose to take part in the research, the average age was 69.17 years, 57% were female, and 70% were a partner or spouse caregiver. In most cases, family did not want to participate in the research as they preferred to have the respite provided by the Nav-CARE volunteer.

### Factors influencing feasibility, acceptability, and sustainability

**Figure 1.**
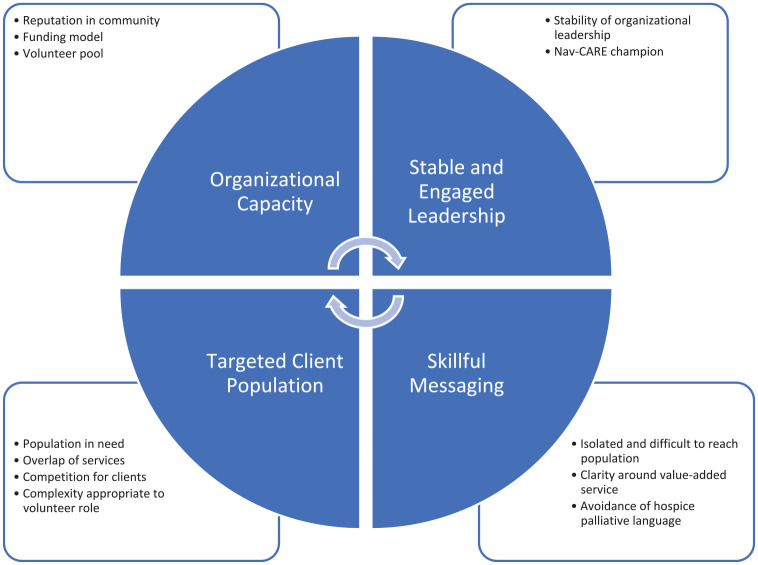
Factors influencing Nav-CARE development.

Key informants identified a number of factors that influenced the feasibility, acceptability, and sustainability of the Nav-CARE program within their organization (Figure 1). These included organizational capacity, stable and engaged leadership, a targeted client population, and skillful messaging (Table 3).

**Table 3.** Themes and additional sample quotes.

Themes	Sample quotes
Organizational capacity	*The community knows we are out there but they don’t necessarily know what we are capable of doing. (VC)* *We all seriously thought we were going to be flooded by clients from the community which would have overwhelmed us but that didn’t happen. (VC)* *We need more support from healthcare providers but also from community service type organizations. (VC)* *Our advisory committee had broad representation who were willing to be champions. (ED)*
Stable and engaged leadership	*It is important to have someone explicitly focused on Nav-CARE. (VC)* *I think if I’m really honest and about how it happened here is that it didn’t help that we had a couple of staff changes throughout the program. You just lose impetus. (VC)* *If you have one person in charge right from the beginning that makes a difference. (VC)*
Targeted client population	*Normally our referrals are more near end of life so this is a different population. (VC)* *We were on the radar of physicians and the social worker and so those referrals really flowed. (VC)* *We are an urban center and there are already a lot of services for older persons in our community. (ED)*
Skillful messaging	*Nav-CARE [team] really helped us um with the language of how to promote us and I think it would’ve been much harder had we not been a part of the study. (VC)* *It’s just going to take time to build up knowledge of the program within our city. (VC)* *We still have to talk about Nav-CARE a lot, just for people to get their head around how it works. (VC)*

ED, executive director; VC, volunteer coordinator.

#### Organizational capacity

Organizational capacity included the reputation of the organization in the community, the funding model, and the current pool of volunteers. Organizations that had a successful implementation were typically already well-integrated into their communities and had a reputation for developing and sustaining high-quality programs. Furthermore, these organizations had a funding model that provided some leeway for innovative, new programs as opposed to only funding programs that provided care to an end-of-life population. They also had a pool of volunteers who were interested in working with clients who had less-intensive needs than those at end-of-life. Organizations in which Nav-CARE was less feasible were overcommitted in their current programming, ‘I think the possible tension is taking on a new program without a full assessment of is this really truly doable for the staff people who are going to have to pull this together and make it work?’ (VC). Other organizations had funding models that only included reimbursements for patients who were designated palliative by a physician, ‘We were only funded for patients who have been designated palliative so that was difficult for us’ (VC). Furthermore, others were primarily known for offering palliative beds rather than community-based services, and this made integration of Nav-CARE difficult. ‘Although we have been operational for 5 years, we are still challenged to make ourselves known in the community’ (VC). For example, it could be difficult for urban-based hospice societies to implement Nav-CARE if their primary function was to provide beds for end-of-life and they had few community-engaged programs.

#### Feasibility

Nav-CARE feasibility was also determined by stable and engaged leadership. In several implementation sites, the senior leadership changed during the implementation period, and in all these sites, Nav-CARE was de-prioritized under the new leadership. ‘Have consistent, consistent leadership right from and start and the executive director, coordinator, and volunteers need to move forward together. Everything flows from that’ (ED). In these cases, the program was either canceled or simply not allocated enough resources to be implemented properly. The organization-based VC also played a critical role. Program implementation was only feasible if this VC was familiar with the goals of the program, committed to seeing it succeed, and had sufficient dedicated time to establish the program. ‘My position is already full time and busy with a lot of things so that you can’t devote as much to things as you would like so if Nav-CARE was the main focus of someone’s portfolio it would make a difference’ (VC). Program implementation was less feasible if there was turnover in this position, if the coordinator was not clear on their role and responsibilities, or if this job was simply added to a pre-existing role and the coordinator did not have the capacity to support the program. ‘This is not a program that you can complete off the side of your desk’ (ED). Commitment on the part of all key stakeholders was critical to the successful establishment of the program.

#### Acceptability

The acceptability of the Nav-CARE program to potential clients was determined by having a clearly identified target population. Nav-CARE seeks to serve persons who are experiencing declining health using an upstream palliative approach to care. ‘The clientele of seniors who are not palliative are a really good target audience’ (VC). In targeting that population, it was important for organizations to determine what other organizations in the community were also providing services to this population to ensure that there was no confusion or overlap in services, thus inadvertently setting up a competition for clients. ‘We struggled to find our niche for tapping into the further upstream population because we’re well known as a hospice program’ (VC). Hospice societies were faced with the challenge of ‘rebranding’ their services to reach a clientele that might not normally be served by hospice. This needed to be done in a way that was acceptable to other organizations and initiatives in the community. Organizations that found an acceptable niche often targeted populations who had few or no existing services in their community (e.g. long-term care residents and dialysis patients discontinuing treatment). ‘There are just so many people living at home who have a chronic condition, they have lots of needs but they don’t yet qualify for community services’ (ED). However, in finding this target population, it was also important for organizations to consider the role of the volunteer in relation to the potential complexity of the client. It was not uncommon in this study for organizations to receive referrals for clients whose needs were beyond what was considered appropriate for volunteers, most notably those clients with complex mental health issues.

Once the target population was determined, the acceptability of Nav-CARE to clients was determined by the messaging used to recruit the population. This was a difficult client population to recruit largely because they often lived alone and isolated in the community, with no one aware of the needs they were experiencing. To recruit them successfully, organizations had to identify these clients, be clear about the value-added nature of the services, and avoid hospice or palliative language as most clients did not see themselves within this category. ‘The message was very clear about what we were offering but sometimes people just didn’t see that they needed it’ (VC). Potential clients were lost if on the initial telephone contact the words hospice/palliative were used, as is often the case with hospice palliative care intake lines. ‘People are put off because we are connected with hospice. So I am wondering if potential Nav-CARE clients are thinking ‘oh good grief I’m not dying yet!’’ (VC). Two of the three nonhospice organizations were unable to recruit any clients; the third organization used pre-existing clients, layering Nav-CARE onto currently existing services. Most recruitment was done through word of mouth as organizations were concerned that they could not manage the large number of clients that they anticipated would take advantage of the service.

At the conclusion of the intervention, in regards to sustainability (programs committed to continuing Nav-CARE), seven sites were sustainable, two sites were unsure (related to the COVID-19 pandemic resolution), and six were not sustainable. In all of the nonhospice organizations, Nav-CARE was unsustainable. Two were unable to implement the program because of difficulties in finding volunteers or clients, and one was able to implement but not sustain the program. Organizations chose to discontinue the program for the following reasons: a change in the direction from the Board, insufficient resources to continue the program, inability to recruit clients, or overlap with existing community-based services. Sustainable programs were able to integrate the Nav-CARE program relatively seamlessly into their current roles and programs, and in some situations, found that having the new program allowed them to generate additional funding from other philanthropic organizations.

### Effectiveness of the Nav-CARE training

The effectiveness of the training in preparing volunteers was measured through self-report using a competency questionnaire. Volunteers overall reported good self-efficacy on the majority of items (see Supplementary Table S1). Self-perceived competency scores (*n* = 86) immediately after the education (T-1) yielded means on the 32 items of 3.54 to 4.6 on a 6-point scale (0 = not at all confident; 5 = very confident). Self-perceived competency scores (*n* = 31) at 6 months posttraining (T-2) yielded means on the 32 items of 3.10 to 4.39 on a 6-point scale. Self-perceived competency scores (*n* = 23) at 12 months posttraining (T-3) yielded means on the 32 items of 2.82 to 4.39 on a 6-point scale. No competency items at any time point indicated modes of less than 3 (indicating feeling incompetent). After volunteers had worked with clients for 12 months (i.e. T-3) competency items on which greater than 15% (*n* = > 5/23) of volunteers rated themselves as incompetent (0-2) included the following: creating linkages to local leaders, professionals, and resources; developing plans reflective of client/family needs and concerns; coordinating access to needed services; assessing client/family service usage; and facilitating beginning discussion with client/family about advance care planning and goals of care.

An important factor to consider in relation to these scores was the degree of ongoing education and mentorship received by these volunteers over the intervention period. The research team offered monthly mentorship teleconferences and continuing education opportunities (*n* = 25 sessions) on topics such as bereavement, COVID-19, and spiritual care. A total of 137 volunteers attended over the 25 sessions. An average of four volunteers participated in each session in 2019 and an average of seven volunteers participated in each session in 2020. Beyond that, some site-specific VCs were providing ongoing mentorship to their volunteers while other coordinators did not. In summary, the Nav-CARE training was effective in preparing volunteers for their role in Nav-CARE. Competencies that overlapped with those of healthcare providers could be emphasized more in the education to ensure that volunteers are clear about their role.

### Impact of Nav-CARE program on clients and family

Clients indicated a number of QOL concerns on their initial intake form that prompted their participation in the Nav-CARE program. These included physical concerns such as pain, mobility challenges, and sleeping problems; emotional concerns such as anxiety, sadness, and healthcare-related stress; social concerns such as family conflict, loneliness, and no one to confide in; environmental concerns such as inappropriate living arrangements, difficulties with activities of daily living, and financial challenges; and existential/spiritual concerns such as an uncertain future, lack of confidence in abilities, and disconnection from spiritual communities.

The semistructured interviews (*n* = 107) conducted with clients and volunteers provided specific examples of volunteer interventions that enhanced QOL for clients under the domains of Connecting, Advocating, Resourcing, and Engaging.

Connecting: ‘I just find her such a caring person. I don’t know how to explain it better than that. She makes me feel better just being around. It is nice to talk to someone about current events or just about silly things that have happened to us. She’s become a friend’ (Client). The impact of volunteers on clients’ feelings of connection and social support was prominent in the interviews. Clients described their relationships with their volunteers as good, comfortable, trusting, enjoyable, easy, intimate, and sharing. When relationships were experienced as difficult it was usually described as a mismatch in personalities that led to awkwardness. Activities that contributed to a supportive connection included fun outings (e.g. coffee, entertainment), shared daily tasks (e.g. grocery shopping), casual conversation that went beyond the client’s health concerns, and acknowledging one another on special occasions. Emotional support was particularly important to clients, particularly as it related to health issues, grief, and existential concerns. Volunteers provided emotional support in the form of distraction, discussion, listening to concerns, offering encouragement, working through complex decisions, and being available when needed. What was particularly important about this emotional support was that it happened outside of the family and so clients felt that there was less of a burden on their loved ones. In addition, clients explained that sometimes having someone outside the family was a good way to lighten up the mood, and shift their focus away from their illness experience.

Advocating: ‘I chose to have a navigator for one reason really, I needed an advocate and I’d used a friend and it was too much. My friend works very hard and has children and so it was too much for her. I needed to find someone who could advocate on my behalf’ (Client). Volunteers advocated on behalf of their clients in a number of ways. They supported clients during healthcare appointments, facilitated self-advocacy, and represented their needs to external organizations. Appointment support included preparation for appointments, moral support during appointments, and filling out health-related paperwork. Self-advocacy roles included helping clients identify important questions to ask healthcare providers. Volunteers also advocated for their clients at an organizational level. Examples of volunteer advocacy with external organizations included attending community meetings to become more knowledgeable about what existed, and writing letters to key decision-makers about community services that were missing or of poor quality. This advocacy role was particularly important if families were not present to help.

Resourcing: ‘I have learned to deal with some things and to accept more help. She connected me to a program in the community that now comes out to help me with cleaning. She also helped me to find low-income housing. I didn’t even know such places existed’ (Client). Participants reported examples of helping clients to identify and access resources that improved their QOL. These included assisting with getting appointments, goal setting to identify what they needed, transportation, food services, downsizing the client’s home, dealing with government services, suggesting healthy activities, and referring clients to community services. Clients spoke of their confidence in the volunteer’s accessing and resourcing abilities. The consensus was that their volunteers were willing and able to locate resources in a timely manner. Clients suggested that they could have benefited from more assistance from the volunteer in this area.

Engaging: ‘She helps me out with my goals for the week. She gets my appointments and gets me involved in exercise programs. She tries to connect me to the community’ (Client). Clients and volunteers described activities that they had done together to support engagement. These activities were designed to re-engage clients in activities that they had previously found enjoyable. The volunteer role was to determine what was important to the client, make suggestions, and then provide peer support. Activities included games, doing art, playing music, and going on outings. Volunteers also sought to engage clients with community supports such as local churches or senior’s centers. In addition, some volunteers engaged in goal-setting exercises with clients to promote wellness. Goals could include personal grooming, exercise, healthy eating, or intellectual development (e.g. taking courses). Clients described how their volunteers helped their engagement in goal-setting activities. This was done through fostering hope, establishing a sense of accountability through regular check-ins, and decreasing barriers to participation. Some volunteers took on the role of facilitators by providing tangible support such as worksheets.

Client participants in this study reported statistically significant gains on several items on the engagement questionnaire. They reported statistically significant improvements in the feeling they have someone to turn to and knowing the services available to help them in their community at the 6-month measurement interval, and in their ability to be involved in the things that are important to them and confidence in taking care of their illness at the 12-month measurement interval (Table 4).

**Table 4.** Engagement questionnaire results for older persons.

Item	T-1, *n* = 50, mean (SD)	T-2, *n* = 28, mean (SD)	T-3, *n* = 27, mean (SD)
I feel I know the services available in my community to help me	2.84 (0.10)	2.21 (0.96)*	2.56 (0.97)
I feel like I have people to turn to when I need help	2.60 (1.26)	2.11(1.03)*	2.44 (1.01)
I feel lonely	3.12 (1.32)	3.15 (1.00)	3.11 (0.89)
I feel I can be involved in the things that are important to me	2.94 (1.20)	3.04 (1.34)	3.42 (1.24)*
I feel I have someone I can talk to about the things that are troubling me	2.70 (1.25)	2.29 (1.27)	2.63 (1.15)
I feel confident in making decisions about my life changes I know where to get information about my illness	2.10 (1.18)	1.93 (0.86)	2.15 (1.03)
	2.18 (0.91)	2.04 (0.96)	2.21 (1.06)
I feel confident in taking care of my illness	2.43 (0.89)	2.38 (0.98)	2.50 (1.06)*
I am confident contacting someone when I have a health problem	1.96 (0.97)	2.04 (1.19)	2.29 (0.96)
I understand the information given to me by my doctor	1.9 (0.93)	1.67 (0.78)	2.00 (0.78)
I feel confident making decisions about my health and healthcare	2.00 (0.97)	1.74 (0.81)	2.08 (1.02)
I feel confident communicating my needs and wishes to my doctor	1.82 (0.98)	1.93 (0.96)	1.79 (0.83)

* Statistically significant change *p* < 0.05.

In addition to the open-ended questions in the interview, clients were asked specific questions related to the Nav-CARE intervention When asked whether Nav-CARE had affected the experiences of family or friends, 66.7% reported a positive impact related to respite from physical and emotional care, improved family communication, and a more positive effect within the family. When asked whether Nav-CARE had cost or saved them money, 45.4% stated that Nav-CARE had saved them money through practical assistance; no participants said the program cost them money. When asked whether Nav-CARE had changed in any way their visits with healthcare providers, 20% suggested that they gained more confidence to be involved in their care; the remainder suggested there was no change. When asked whether Nav-CARE was important, 82.4% rated the program between 6 and 10 on a 10-point scale and when asked how satisfied they were with the program 95.5% rated the program between 6 and 10 on a 10-point scale. When asked whether Nav-CARE had improved their QOL, 88.2% indicated improvement, while the other 11.8% reported no change.

However, the QOL (SF12v2) scores did not reflect this positive change. Bodily pain score showed a statistically significant increase by about 7.14 units at T2 (*p* = 0.196) compared with baseline and decreased to about 9.91 units at T3 (*p* = 0.036). These scores indicate that bodily pain was the worst at the third time point as compared with baseline scores. All the other QOL functions were similar to the baseline, and none of the changes over time was statistically significant. The physical component score (PCS) and mental component score (MCS) also did not show any significant changes over time (Table 5). In summary, older persons reported positive benefits from having a Nav-CARE volunteer in the qualitative interviews. Specific benefits were reflected in improved scores on items on the engagement questionnaire; however, there were no statistically significant improvements in QOL scores.

**Table 5.** QOL results for older persons.

	T1, mean (*n*)	T2, mean (*n*)	T3, mean (*n*)	Comparing T2 with T1	Comparing T3 with T1
Physical functioning	18.88 (49)	20.54 (28)	21.30 (27)	0.622	0.559
Role physical	30.92 (49)	29.46 (28)	31.78 (27)	0.601	0.829
Bodily pain	43.37 (49)	53.70 (27)	30.56 (27)	0.196	**0.036** *
General health	32.86 (49)	34.82 (28)	33.85 (26)	0.404	0.649
Vitality	31.12 (49)	30.56 (27)	31.48 (27)	0.813	0.977
Social Functioning	44.90 (49)	53.70 (27)	40.00 (25)	0.454	0.325
Role emotional	56.47 (49)	57.31 (28)	48.54 (27)	0.803	0.180
Mental health	53.06 (49)	57.31 (28)	57.87 (27)	0.305	0.203
Physical component score	33.03 (49)	33.66 (27)	32.24 (27)	0.763	0.666
Mental component score	43.58 (49)	45.60 (27)	43.15 (27)	0.971	0.403

* Statistically significant *p* < 0.05.

### What was the impact of being a Nav-CARE volunteer?

In the interviews, volunteers spoke of the benefits they received from participating in the Nav-CARE program including the ability to make a difference in the lives of others, the opportunity for ongoing learning, and the sense of connection with clients. Volunteers expressed a sense of satisfaction in making a difference in the lives of their clients. ‘I feel like I am really helping people and bringing a little ray of sunshine’ (Volunteer). This was particularly relevant when they saw themselves as part of a bigger compassionate community movement. ‘I think my favorite part was knowing that this can develop into a bigger picture that’s creating a healthier community’ (Volunteer). Volunteers also appreciated the opportunity for ongoing learning provided by the initial education, the mentorship sessions, and their ongoing experiences with helping clients. ‘I feel like I have learned a lot about what is out there in relation to services’ (Volunteer). ‘I believe wholeheartedly in life-long learning and so I have attended all of the training sessions’ (Volunteer). Benefits also included a sense of relationship and connection. ‘The part I like most is getting to know people and to hear about their life adventures’ (Volunteer). Another volunteer spoke of how being involved in Nav-CARE was a way for them to be involved in the community. ‘It has been a way to become more connected to my community’ (Volunteer). Overall, volunteers suggested that being involved in Nav-CARE was at times as much for their benefit as that of the clients and how important it was to make a meaningful contribution. ‘It enriches my life as least as much as anything I do for anyone else’ (Volunteer).

Overall volunteer satisfaction was reflected on the satisfaction questionnaire. The higher the scores, the higher the satisfaction. Item means on the questionnaire at 6 months ranged from 3.07 to 4.46 on a 5-point scale. Similar satisfaction was reflected at 12 months with item means ranging from 3.21 to 4.48 on a 5-point scale (Table 6).

**Table 6.** Subdomains for volunteer satisfaction measure.

Subdomain: total possible score	Time 1, *n* = 32, mean (SD)	Time 2, *n* = 17, mean (SD)
Orientation: 20	14.88 (3.68)	16.14 (3.39)
Training: 40	29.64 (5.22)	31.62 (5.36)
Feedback: 45	34.06 (9.23)	33.91 (6.82)
Communication: 35	25.81 (5.65)	25.64 (4.13)
Social: 20	13.26 (2.14)	13.40 (2.89)
Valued: 55	45.83 (6.90)	45.22 (5.83)

Subdomains that produced the highest satisfaction scores were related to orientation, training, and communication. Subdomains that produced the lowest satisfaction scores were related to the social aspects of their role that included connecting with volunteers and others within their organization (see Supplementary Table S2 for item scores).

The SF12v2 QOL domains for the volunteer data did show changes over time (Table 7). Physical functioning scores were highest at the baseline and showed a decrease over time. The decrease in physical functioning from baseline to T2 was about 7.56 units (*p* = 0.026) and for T3 was about 4.60 units (*p* = 0.146). Role physical score increased to about 0.60 units for T2 (*p* = 0.831), however, this increase was not statistically significant. For T3, the role physical score decreased by 4.25 units compared with baseline and this difference was statistically significant (*p* = 0.022), indicating poor role physical at T3 compared with baseline. PCS was very similar for baseline and T2, and for T3, the PCS was about 1.63 units less as compared with baseline (*p* = 0.078) indicating poor PCS at T3. MCS showed a slight increase over time; however, this difference was not statistically significant. In summary, volunteers reported their role in Nav-CARE as satisfying and meaningful and appreciated the opportunities for further learning. QOL results suggested some decrease in physical functioning and role scores.

**Table 7.** QOL results for volunteers.

	T1, mean (*N*)	T2, mean (*N*)	T3, mean (*N*)	Comparing T2 with T1	Comparing T3 with T1
Physical Functioning	88.13 (85)	81.50 (50)	84.85 (33)	**0.026** *	0.146
Role physical	82.94 (85)	84.69 (49)	76.89 (33)	0.833	**0.022** *
Bodily pain	83.53 (85)	84.69 (49)	87.12 (33)	0.706	0.242
General health	81.90 (84)	81.20 (50)	77.19 (32)	0.362	0.194
Vitality	69.05 (84)	68.88 (49)	67.42 (33)	0.284	0.474
Social functioning	89.88 (84)	93.35 (49)	90.91 (33)	0.489	0.774
Role emotional	91.03 (85)	89.26 (49)	92.80 (33)	0.294	0.339
Mental health	79.51 (84)	77.55 (49)	79.17 (33)	0.116	0.991
Physical component score	52.77 (84)	52.49 (49)	51.45 (33)	0.637	0.078
Mental component score	54.70 (84)	54.46 (49)	55.21 (33)	0.373	0.313

* Statistically significant *p* < 0.05.

## Discussion

The purpose of this study was to scale out a social innovation called Nav-CARE while conducting a mixed-method evaluation to build further evidence of the intervention. Scaling out, or ‘impacting greater numbers’^3^ (p. 3) of participants is important before the scaling up work of law and policy. This study provided additional evidence about the importance of training and mentorship for Nav-CARE volunteers. Self-reported competency assessments indicated that volunteers could use additional education in areas where their role intersected with that of healthcare providers. These areas included identifying community resources, assisting with decision-making, discussing advance care planning, and creating linkages to local leaders and resources. These findings were validated through the volunteer satisfaction measures; satisfaction scores were lower in domains such as understanding the medical and social needs of clients and knowing the bigger picture of palliative care in the community. Volunteers also provided lower satisfaction scores related to their connection to their volunteer organization. Intraorganizational support was an important part of volunteer satisfaction and perceived competence. Future development of the Nav-CARE program should include a more explicit focus on intraorganizational mentoring.

This study further illustrated the resourcefulness and creativity of these volunteers as they performed the basic Nav-CARE roles of connecting, advocating, resourcing, and engaging. Such data provide important evidence about the capacities of volunteers beyond that of friendly visiting. The role these Nav-CARE volunteers performed was indicative of best practices for programs that seek to provide supplementary support for older persons; such programs have four main outcomes: enriching relationships, supporting autonomy and control, enhancing knowledge, and improving mental health.^33^ Volunteers too described benefits they experienced as a result of providing services to clients including ongoing learning, making a meaningful contribution to the life of someone else, and enjoying the companionship of their client. Although physical and role functioning QOL scores for these volunteers declined at some time points, it is important to remember that some of these data were collected during the time when COVID-19 lockdowns were in place.

Findings from this study indicated positive impacts on clients. Similar to findings in our previous studies, clients described how volunteers helped them with social support, advocacy, information-finding, goal setting, decision-making, resource access, and participation in meaningful activities.^23,24^ When asked whether Nav-CARE had improved their QOL, 88% of clients reported improvement. These benefits were not reflected in improvements in QOL scores over time. It is possible that volunteer interventions are not intense enough to influence the global scores that are reflected in overall QOL measures; although study participants were able to isolate that contribution in their qualitative reflections.

Developing a deeper understanding of what influences the Nav-CARE development and sustainability at the organizational level was an important outcome of this study. In previous studies exploring implementation factors affecting Nav-CARE, we identified specific barriers and facilitators of program development. These barriers and facilitators related to public knowledge and perceptions of palliative care; social and financial organizational capital; and skilled leadership.^24,28^ In the study reported in this article, we were able to analyze those factors across diverse contexts to develop a more nuanced understanding of the factors that must be in place to produce a robust and sustainable program. These four factors were organizational capacity, stable and engaged leadership, a focused client population, and skillful messaging. Difficulties in implementation across contexts could be traced back to at least one of these four factors. Furthermore, we learned that these factors had varying impacts depending on the organizational context. For example, urban hospices that had hospice beds were often viewed by the community as the place where people go to die which in turn made skillful messaging of the Nav-CARE program more important. In contrast, rural hospices were already well known for their community-engaged programs and so messaging this new program may have been easier. Although the project was unable to gather in-depth implementation data the four factors identified in our study align with CFIR domains and constructs affecting successful implementation (e.g. the degree to which an organization is networked with other organizations, leadership engagement, available resources, and engaging others in implementation through marketing).^25^ Understanding how these four factors interact within a given context can support organizations to implement strategically.

Another important finding was the degree to which these organizations were struggling collectively to realize their vision of developing community-engaged programs in keeping with the compassionate community/public health approach to palliative care – that ‘bottom-up’ approach to care that is at the foundation of high-quality palliative care.^8,9,34^ Each organization was striving to provide high-quality services within a broader context that required significant attitudinal shifts within their community to enable them to realize their vision. We will discuss this context in terms of the other two factors critical to social innovation: scaling deep and scaling up.

### Scaling deep: influencing relationships and value

The goal of scaling deep is to impact cultural roots through influencing relationships, cultural values, and beliefs.^3^ The beliefs that often influence the success of hospice and palliative care volunteer organizations are long-standing ideas about the appropriate role for, and value of, volunteer hospice services. Although hospice societies have a long-standing tradition within palliative care, they experience a number of barriers to receiving referrals in the Canadian context including volunteers not being part of the formal healthcare team, patient, and family who may not be ready to be involved with an organization that cares for the dying, and lack of knowledge about the role and training of volunteers.^5^ Too often, hospice services are believed to be only appropriate for those who are actively dying, and the impact of volunteer interventions is underappreciated. In addition to having to negotiate these existing barriers, hospice societies implementing Nav-CARE were now seeking to serve an upstream palliative population and to provide QOL navigation. This required rebranding and marketing a new image of who could benefit from their services. This new image was complicated when there was an overlap in services with other community-based organizations that also served a population living with declining health (e.g. disease-specific organizations and senior centers that seek to serve the vulnerable). This potentially led to a competition for clients, a competition that was difficult for hospice societies when there was so much public stigma around death and dying. Societies that were already scaling deeply in their communities by educating stakeholders about the value of hospice services had the most success with Nav-CARE implementation.

A recent review of the evidence on public health/compassionate community approaches to palliative care highlighted important values and beliefs that must shift within society for these programs to be successful: viewing responsibilities around death as a shared and negotiated social process; understanding that important knowledge is not just professional but held within the community; learning to communicate using a ripple approach; and focusing on network building. Furthermore, two realities of palliative care make the adoption of these values and beliefs challenging. The first is the overemphasis on professional end-of-life care to the detriment of community-based approaches. The second is the discrepancy between how dying is perceived by palliative care providers and by the public. This review concluded that strong leadership is critical to helping address these tensions.^35^

In this study, organizations that had a successful Nav-CARE implementation were already well connected to their community having built a strong network of relationships. They had carefully negotiated the appropriate role of the volunteer in relation to formal healthcare, and they had strong and consistent leadership to champion the program. In summary, as an organization, they were already leveraging the values and processes that supported a public health approach. However, this challenging and labor-intensive work was being done at the individual organizational level, which leads to the final question of what policy issues need to be considered to further scale up volunteer-led interventions such as Nav-CARE.

### Scaling up: influencing policy

At the conclusion of the intervention, approximately half of the Nav-CARE sites were sustainable, which is the Nav-CARE program continued beyond the 1-year intervention period. Participants described a number of challenges related to sustainability, the majority of which traced back to a lack of resources. Although the day-to-day running of the Nav-CARE program once it was established was not labor-intensive, doing the public education regarding the program and the recruitment of clients was. The leadership function of doing the important work of scaling deep in the community as described above required dedicated coordinator time. The challenge of finding resources is endemic to nonprofit hospice societies which must fundraise continually to support their efforts and do the delicate balancing act of community need and organizational capacity.

Over a decade ago, Senator Sharon Carstairs in her report to the Senate recommended that the delivery of palliative care, whether in institutions or at home, be supported by volunteers to maximize effectiveness.^36^ The recent Framework on Palliative Care in Canada^1^ and the report by the Canadian Society of Palliative Care Physicians^37^ reiterated the important role of volunteers in providing community support for persons living with a palliative diagnosis. Significant progress has been made in building volunteer hospice palliative care in Canada. Hospice societies in Canada have important advocacy bodies at the provincial and national levels and have built broad-based advocacy coalitions (e.g. Canadian Hospice Palliative Care Association and the Quality End-of-Life Coalition). Volunteer training is accessible and of high quality, for example, Canadian Hospice Palliative Care Association^38^ directed by volunteer competency documents^39^ that clearly outline the role and required preparation. Much foundational work has been done to support a robust volunteer network. However, these societies must still spend substantial resources raising funds to support their efforts.

The reputation of being a volunteer society belies the resources that are required to make significant contributions to community-based palliative care, particularly if the public health/compassionate community approach is indeed everyone’s responsibility. High-quality programs rely on robust organizational capacity, even if the services are largely provided by volunteers. Building community capacity in a public health/compassionate community approach requires dedicated leadership. Excellent theoretical frameworks^40^ and toolkits^41,42^ are now available to support this approach, but these implementation strategies require dedicated funds and engaged leadership. If volunteer hospice and palliative care organizations are central to realizing this approach, then changes in policy are required to assist them in doing this important work. Indeed, such pragmatic considerations of how to actually build this community capacity seem to be missing from the current conversation.

An important policy consideration is determining which societal organizations might best do this work. Should these community-based interventions be part of the formal health and social care systems? A recent report from the National Academies Press^43^ recommends that while those in healthcare are well poised to identify older persons at risk for social isolation and loneliness, the responsibilities for addressing this cannot reside within formal healthcare, it is beyond their scope. The same argument could be made for social care systems. The challenge then lies in building these programs outside of formal health and social care systems while ensuring adequate funding and accountability and connecting them strategically to health and social care to maximize the impact.

Although it is beyond the scope of this article to address the specific policy work that would be necessary to realize this ideal, there are certain steps that could be considered. First, in addition to the existing Canadian Palliative Care Framework^1^ and Action Plan,^44^ national quality indicators for palliative care would provide an important accountability framework.^45^ Canada currently has no standardized quality indicators for end-of-life care that occurs in the home.^46^ Such standardized indicators should include volunteer involvement which could then be embedded into care and mapped over time. Such a step would help to offset the challenge of overemphasizing the palliative care delivered by professionals while making visible the commitment to public health/compassionate community approaches. Second, dedicated baseline funding for these societies would allow them to provide important leadership for the scaling deep work necessary to realize the public health/compassionate community approach. Such funding would be provided within an accountability framework for services provided. Without clearly delineated responsibilities within a quality framework and adequate funding, it will be difficult for volunteer societies to provide the necessary leadership to realize the compassionate community approach that is part of the goal for high-quality palliative care.

The findings of this study have important limitations. The public-health physical distancing policies that arose from the COVID-19 pandemic meant that volunteers who were active as of March 2020 had to switch to virtual-only visits with their clients. This change was impactful for clients and volunteers, resulting in the loss of the face-to-face relational building that is so foundational to the effectiveness of the program.^47^ Furthermore, it became even more difficult for organizations to recruit new clients. Seven of the 15 organizations were still within their 1-year intervention period, and two of those were just beginning the intervention period. Some volunteers switched to virtual visits, but others stopped meeting with their clients because clients did not want virtual visits. An additional limitation was that the scale of the project did not allow for the collection of detailed implementation data that is typically required of the CFIR framework.^25^ Despite these limitations, this scale-out study replicated findings from previous studies^23,24^ and further developed our knowledge of the feasibility, acceptability, and impact of the intervention.

## Conclusion

This scale-out study of a volunteer-navigation intervention called Nav-CARE provided insights into feasibility, acceptability, and sustainability across contexts. Although a number of organizations that participated in this study were able to develop robust and sustainable Nav-CARE programs, it was largely due to strong intraorganizational leaders who were able to address some of the barriers that have been endemic to realizing a compassionate community approach, specifically those values and beliefs that constrain the role and image of community-based hospice palliative care. The study further provided insights into the impact of being a Nav-CARE volunteer, and the important impact volunteers can have on the lives of older persons living with declining health. Volunteers described benefits they encountered such as engaging in ongoing learning, feeling as if they were making a meaningful contribution, and enjoying the relationships developed with clients. Clients in this study stated that having a volunteer improved their QOL through enriched relationships, deeper engagements, and better access to resources. Although the physical distancing requirements of the COVID-19 pandemic interrupted the fidelity of the intervention, the study provided important information about the organizational factors that support such public health/compassionate community approaches to care. The pandemic further provided an opportunity to develop a model on virtual volunteering which has become a regular part of the Nav-CARE training. Future scale-deep and scale-out work is required to assist those community-based hospice palliative care societies that are pushing forward this important approach.

## Supplemental Material

sj-docx-1-pcr-10.1177_26323524221095102 – Supplemental material for Scaling out a palliative compassionate community innovation: Nav-CARE Click here for additional data file. Supplemental material, sj-docx-1-pcr-10.1177_26323524221095102 for Scaling out a palliative compassionate community innovation: Nav-CARE by Barbara Pesut, Wendy Duggleby, Grace Warner, Sunita Ghosh, Paxton Bruce, Rowena Dunlop and Gloria Puurveen in Palliative Care and Social Practice

sj-docx-2-pcr-10.1177_26323524221095102 – Supplemental material for Scaling out a palliative compassionate community innovation: Nav-CARE Click here for additional data file. Supplemental material, sj-docx-2-pcr-10.1177_26323524221095102 for Scaling out a palliative compassionate community innovation: Nav-CARE by Barbara Pesut, Wendy Duggleby, Grace Warner, Sunita Ghosh, Paxton Bruce, Rowena Dunlop and Gloria Puurveen in Palliative Care and Social Practice
