# Supplementary material for: Scaling out a palliative compassionate community innovation: Nav-CARE
Source: Palliat Care Soc Pract. 2022 May 13;16:26323524221095102. doi: 10.1177/26323524221095102 (PMC9112317; doi:10.1177/26323524221095102)
Supplement: sj-docx-2-pcr-10.1177_26323524221095102 – Supplemental material for Scaling out a palliative compassionate community innovation: Nav-CARE [file sj-docx-2-pcr-10.1177_26323524221095102.docx]

Supplementary Material

Table S1. Volunteers reporting less than confident on self-efficacy items.

|  | ***Not Confident (0-2)*** | | | ***Confident (3-5)*** | | |
| --- | --- | --- | --- | --- | --- | --- |
| Items | ***T-1 N = 86*** | | ***T-2 N=31*** | | ***T-3 N=23*** | |
|  | ***N < 3*** | ***%*** | ***N<3*** | ***%*** | ***N<3*** | ***%*** |
| 1. Know when to access my volunteer coordinator | 1 | 1 | 2 | 5.4 | 1 | 2.7 |
| 1. Assess client/family quality of life concerns | 3 | 3.4 | 1 | 2.7 | 2 | 5.4 |
| 1. Identify client/family values and beliefs regarding end of life issues | 5 | 5.7 | 2 | 5.4 | 3 | 8.1 |
| 1. Prioritize with client/family concerns across quality of life areas | 3 | 3.4 | 4 | 10.8 | 3 | 8.1 |
| 1. Determine client/family preferred engagement in decision making and self-navigation | 4 | 4.6 | 4 | 10.8 | 5 | 13.5 |
| 1. Identify client/family knowledge and perception of available options and community resources | 6 | 6.9 | 3 | 8.1 | 2 | 5.4 |
| 1. Determine if there is a need for community resources and services | 10 | 11.5 | 6 | 16.2 | 3 | 8.1 |
| 1. Identify client/family needs for resources | 9 | 10.3 | 3 | 8.1 | 3 | 8.1 |
| 1. Assist family caregivers in care provision decisions | 7 | 8.0 | 5 | 13.5 | 4 | 10.8 |
| 1. Identify barriers to needed resources | 6 | 6.9 | 3 | 8.1 | 3 | 8.1 |
| 1. Advocate to meet client/family needs with healthcare professionals | 6 | 6.9 | 3 | 8.1 | 1 | 2.7 |
| 1. Assist client/family to overcome service access barriers | 5 | 5.7 | 4 | 10.8 | 2 | 5.4 |
| 1. Advise client/family on negotiating for care and services | 7 | 8.0 | 7 | 18.9 | 4 | 10.8 |
| 1. Facilitate strategies for self-navigation | 7 | 8.0 | 6 | 16.2 | 3 | 8.1 |
| 1. Perform an environmental scan for community services, care providers, and events | 7 | 8.0 | 3 | 8.1 | 3 | 8.1 |
| 1. Identify community assets for client/family | 6 | 6.9 | 6 | 16.2 | 3 | 8.1 |
| 1. Identify best-fit for client/family with community resources | 7 | 7.0 | 6 | 16.2 | 4 | 10.8 |
| 1. Create linkages to local leaders, professionals and resources * | 9 | 10.3 | 9 | 24.3 | 7 | 18.9 |
| 1. Assist client/family to build and connect (or reconnect) with networks/connections | 7 | 8.0 | 5 | 13.5 | 4 | 10.8 |
| 1. Identify client/family concern and/or needs | 4 | 4.6 | 2 | 5.4 | 1 | 2.7 |
| 1. Assess client/family need for support | 6 | 6.9 | 1 | 2.7 | 2 | 5.4 |
| 1. Develop plans reflective of client/family needs and concerns * | 7 | 8.0 | 7 | 18.9 | 6 | 16.2 |
| 1. Provide family with caregiving support and resources | 8 | 9.2 | 4 | 10.8 | 5 | 13.5 |
| 1. Coordinate access to needed services * | 5 | 5.7 | 6 | 16.2 | 7 | 18.9 |
| 1. Assess client/family service usage * | 8 | 9.2 | 9 | 24.3 | 6 | 16.2 |
| 1. Facilitate beginning discussion with client/family about advanced care planning (ACP) and goals of care * | 10 | 11.5 | 7 | 18.9 | 6 | 16.2 |
| 1. Assist client/family with access to services for loss, grief and bereavement support | 7 | 8.0 | 7 | 18.9 | 3 | 15.0 |
| 1. Determine effective and appropriate ways to meet client needs | 4 | 4.6 | 5 | 13.5 | 2 | 9.1 |
| 1. Identify level of desired client/family involvement | 7 | 8.0 | 5 | 13.5 | 3 | 13.6 |
| 1. Build capacity with client/family towards their desired level of independence and engagement | 4 | 4.6 | 5 | 13.5 | 3 | 8.1 |
| 1. Promote client/family engagement in decision-making and end of life issues | 9 | 10.3 | 7 | 18.9 | 4 | 10.8 |
| 1. Evaluate continuously the level of client/family engagement in decision-making and end of life issues | 5 | 5.7 | 6 | 16.2 | 4 | 18.2 |

* competencies in which > 15% of volunteers indicated feeling not competent at T-3

Table S2: Volunteer Satisfaction Scores

| ORIENTATION | T1  Mean (SD) | T1  Mode | T1  Range | T2  Mean  (SD) | T2  Mode | T-2  Range |
| --- | --- | --- | --- | --- | --- | --- |
| My orientation to the N-CARE volunteer program was sufficient (e.g., understanding how the program worked). | 4.10 (.99) | 4/5 | 4 | 4.18 (1.02) | 5 | 3 |
| My orientation prepared me to fill out the visit paperwork. | 3.78(1.01) | 4 | 4 | 4.13 (.83) | 4 | 3 |
| My orientation to my assigned client was sufficient. | 3.97 (.99) | 4 | 4 | 4.12 (.93) | 5 | 3 |
| My orientation to community resources was sufficient. | 296 (1.20) | 2 | 4 | 3.56 (1.21) | 4 | 4 |

| TRAINING | T1 Mean  (SD) | T 1 Mode | T1  Range | T2 Mean  (SD) | T2 Mode | T-2 Range |
| --- | --- | --- | --- | --- | --- | --- |
| My training as an N-CARE volunteer was sufficient. | 3.97 (.89) | 4 | 4 | 4.06 (.90) | 4 | 3 |
| Following my training, I felt prepared to be an N-CARE volunteer. | 3.87 (.73) | 4 | 4 | 3.94 (.90) | 4 | 3 |
| My training was offered in a timely fashion. | 4.23 (.76) | 4 | 4 | 4.35 (.70) | 5 | 2 |
| Following my training, I was comfortable to begin work. | 4.10(.79) | 4 | 4 | 4.18 (.53) | 4 | 2 |
| My training prepared me well for the emotional challenges of my position. | 3.80 (.80) | 4 | 3 | 3.94 (.93) | 4 | 4 |
| My training prepared me well for appropriate communication with clients. | 3.93 (.74) | 4 | 4 | 4.00 (.71) | 4 | 3 |
| My training prepared me well for appropriate communication with family members. | 3.36 (.91) | 4 | 4 | 3.79 (.80) | 4 | 3 |
| I do not need any further training to continue work as an N-CARE volunteer. | 2.71 (.97) | 4 | 3 | 3.18  (1.02) | 4 | 3 |

| FEEDBACK / PERFORMANCE | T1 Mean (SD) | T1 Mode | T1 Range | T2 Mean (SD) | T2  Mode | T2 Range |
| --- | --- | --- | --- | --- | --- | --- |
| If I have questions about my N-CARE volunteer work I feel comfortable to ask. | - 1. (.85) | 5 | 4 | 4.41 (.80) | 5 | 3 |
| If I have questions, someone is available to answer them. | 4.29 (.97) | 5 | 4 | 4.18 (.88) | 4/5 | 3 |
| I receive adequate feedback from the N-CARE volunteer coordinator. | 4.17 (1.05) | 4/5 | 4 | 4.06 (.10) | 4 | 3 |
| The feedback I receive from the N-CARE volunteer coordinator is constructive. | 4.17 (1.10) | 5 | 4 | 4.00 (1.03) | 4/5 | 3 |
| The feedback I receive from the N-CARE volunteer coordinator is useful. | 4.18 (1.09) | 5 | 4 | 4.13 (.89) | 4 | 3 |
| I receive adequate feedback from other N-CARE volunteers. | 3.39 (1.08) | 4 | 4 | 3.50 (1.22) | 4 | 4 |
| The feedback I receive from N-CARE volunteers is constructive. | 3.65 (1.07) | 4 | 4 | 3.93 (,83) | ¾ | 2 |
| The feedback I receive from N-CARE volunteers is useful. | 3.70 (1.11) | 4 | 4 | 3.93 (.80) | 4 | 2 |
| Overall, I receive adequate feedback about my performance as an N-CARE volunteer. | 3.70 (.95) | 4 | 4 | 3.81 (117) | 5 | 3 |

| COMMUNICATION | T1 Mean (SD) | T1 Mode | T1 Range | T2 Mean (SD) | T2 Mode | T2 Range |
| --- | --- | --- | --- | --- | --- | --- |
| I am well informed about the medical needs of the N-CARE clients. | 3.63 (1.16) | 4 | 4 | 3.53 (1.06) | 4 | 3 |
| I am well informed about the social needs of the N-CARE clients. | 3.80 (.96) | 4 | 4 | 3.75 (.93) | 4 | 3 |
| I am well informed about broader palliative care issues in my community. | 3.64 (.87) | 4 | 4 | 3.50 (.94) | 4 | 3 |
| I am well informed about events at my volunteer organization. | 4.23 (.82) | 4 | 3 | 4.50 (.63) | 5 | 2 |
| Good communication exists between the NCARE volunteer coordinator, volunteer organization and myself. | 4.23 (.94) | 4 | 4 | 4.06 (.10) | 4 | 3 |
| Good communication exists between the N-CARE volunteer coordinator, other health care professionals and myself. | 3.46 (1.12) | 4 | 4 | 3.36 (.75) | 3 | 3 |
| Good communication exists between other N-CARE volunteers and myself. | 3.54 (1.21) | 4 | 4 | 3.29 (.99) | 4 | 3 |

| SOCIAL CONTACTS | T1 Mean (SD) | T1 Mode | T1 Range | T2 Mean (SD) | T2 Mode | T2 Range |
| --- | --- | --- | --- | --- | --- | --- |
| I know other N-CARE volunteers well. | 2.93 (1.2) | 4 | 4 | 2.94 (.10) | 2 | 3 |
| I would like the opportunity to have more social contacts with N-CARE volunteers. | 3.41 (.83) | 3 | 3 | 3.67 (1.23) | 5 | 3 |
| I know other volunteers in my organization well. | 3.57 (1.14) | 4 | 4 | 3.38 (1.31) | 2 | 4 |
| I would like the opportunity to have more social contacts with my volunteer organization. | 3.37 (.89) | 4 | 3 | 3.38 (1.14) | 4 | 4 |

| VALUE AND RESPECT | T1 Mean (SD) | T1 Mode | T1 Range | T2 Mean (SD) | T2  Mode | T2 Range |
| --- | --- | --- | --- | --- | --- | --- |
| I am a valued member of the N-CARE program. | 4.13 (.85) | 4 | 4 | 4.20 (.78) | 4/5 | 2 |
| I feel my work, as an N-CARE volunteer, is important. | 4.52 (.57) | 5 | 2 | 4.25 (.78) | 5 | 2 |
| I feel my work, as an N-CARE volunteer, is needed. | 4.52 (.57) | 5 | 2 | 4.44 (.63) | 5 | 2 |
| I help improve the quality of life for the NCARE clients. | 4.18(.82) | 4 | 3 | 4.25 (.68) | 4 | 2 |
| My skills as an N-CARE volunteer are used to their full potential. | 3.48 (1.02) | 4 | 4 | 3.44 (.96) | 4 | 3 |
| My volunteer position is what I expected it to be. | 3.57 (1.00) | 4 | 4 | 4.06 (.85) | 4 | 3 |
| I am happy with my volunteer position. | 4.07 (.83) | 4 | 4 | 4.19 (.75) | 4 | 2 |
| Communication with the N-CARE volunteer coordinator is respectful. | 4.61 ((.57) | 5 | 2 | 4.44 (.63) | 5 | 2 |
| Communication with healthcare staff is respectful. | 3.94 (.93) | 4 | 3 | 4.00 (.89) | ¾ | 2 |
| Communication with other hospice staff is respectful. | 4.43 (.60) | 4/5 | 2 | 4.38 (.89) | 5 | 2 |
